# Preliminary Clinical Application of Textile Insole Sensor for Hemiparetic Gait Pattern Analysis

**DOI:** 10.3390/s19183950

**Published:** 2019-09-12

**Authors:** Changwon Wang, Young Kim, Hangsik Shin, Se Dong Min

**Affiliations:** 1 Department of Medical IT Engineering, Soonchunhyang University, Asan 31538, Korea; 2 Department of Computer Science, Soonchunhyang University, Asan 31538, Korea; 3 Wellness Coaching Service Research Center, Soonchunhyang University, Asan 31538, Korea; 4 Department of Biomedical Engineering, Chonnam National University, Yeosu 59626, Korea

**Keywords:** conductive textile, gait, stroke, hemiparetic, real-time monitoring

## Abstract

Post-stroke gait dysfunction occurs at a very high prevalence. A practical method to quantitatively analyze the characteristics of hemiparetic gait is needed in both clinical and community settings. This study developed a 10-channeled textile capacitive pressure sensing insole (TCPSI) with a real-time monitoring system and tested its performance through hemiparetic gait pattern analysis. Thirty-five subjects (18 hemiparetic, 17 healthy) walked down a 40-m long corridor at a comfortable speed while wearing TCPSI inside the shoe. For gait analysis, the percentage of the plantar pressure difference (PPD), the step count, the stride time, the coefficient of variation, and the phase coordination index (PCI) were used. The results of the stroke patients showed a threefold higher PPD, a higher step count (41.61 ± 10.7), a longer average stride time on the affected side, a lower mean plantar pressure on the affected side, higher plantar pressure in the toe area and the lateral side of the foot, and a threefold higher PCI (hemi: 19.50 ± 13.86%, healthy: 5.62 ± 5.05%) compared to healthy subjects. This study confirmed that TCPSI is a promising tool for distinguishing hemiparetic gait patterns and thus may be used as a wearable gait function evaluation tool, the external feedback gait training device, and a simple gait pattern analyzer for both hemiparetic patients and healthy individuals.

## 1. Introduction

The post stroke gait disturbance is one of the major complications that requires a long-term rehabilitation and limits the patient’s activities of daily living [1]. The improved gait function is an important factor in returning to social life and thus is an essential goal of rehabilitation therapy [2]. While 60% to 80% of stroke patients regain their independent ambulation function, many exhibit hemiparetic gait pattern for the rest of their lives due to unilateral neuromuscular weakness leading to gait asymmetry [2,3,4]. The critical consequences of the impaired walking ability after a stroke include a reduction in gait speed, shorter step and stride lengths, and an increased fall risk [5,6]. These residual deficits are mainly caused by muscle weakness and imbalance, decreased weight support on the affected side, and asymmetrical intralimb coordination [7,8]. For the patients with these deviations to regain walking abilities, clinical treatments commonly rely on traditional rehabilitation approaches such as neuromuscular re-education, lower limb strength training, and balance training for weight shifting and gait pattern training. While the patients and healthcare providers strive to seek for more effective gait therapy methods, there are not many long-term remedies or set devices for facilitating these treatment options in clinical settings. 

With fast growing advances in technology, various forms of wearable measuring-recording devices and sensors have been developed for the health care system. For gait rehabilitation, force sensitive resistor sensors (FSR) are one of the most commonly used sensor types for analyzing gait pattern or plantar pressure, but is known to have the disadvantage of being deformed in time as a response to repetitive force applied. Another drawback of the FSR sensor is the need to be constantly calibrated (good for approximately 100 uses) and its inability to distinguish between load changes in respect to the weight bearing level difference generated during walking [9,10]. Another popular type considered for gait biofeedback sensors is piezoelectric material, which has a high impedance, low noise, and susceptibility to electrical interference. However, its weakness is having a limited pressure range and non-linearity in repeated signal output [9]. According to a study by Aqueveque et al. (2018), capacitive sensors can be used for developing an in-shoe device that measures plantar pressure [9]. A capacitive sensor basically consists of two conductive plates that are separated by dielectric material. Modifying the distance between the conductive plates generates a variation of the capacitance and this change in capacitive value can be interpreted as plantar pressure change [9]. This study developed a capacitive insole sensor to analyze plantar pressure and with the aim to provide real-time feedback on the patients’ gait rehabilitation processes. 

The assessment of gait rehabilitation is usually made using clinical scales, but many of the neuromuscular disabilities are still being evaluated manually with analog scales [11,12,13,14,15]. Thus, it is difficult to verify the accuracy in measurement methods, and both the intra- and inter-personal evaluation results have low reliability. A practical device that can quantitatively analyze the characteristics of hemiparetic gait pre and post rehabilitation is substantially needed. A recent study by Ngueleu et al. (2019) stated that, although using pressure-sensing insoles for identification of the step count is a promising approach in gait analysis, the accuracy of the activity monitors in step counting remains limited and that there appears to be no consensus for optimal positioning and the number of sensors for insoles [10]. 

In stroke rehabilitation, the basic gait analysis parameters include the plantar pressure distribution, the step count, the stride time, velocity (or gait speed), the center of pressure (CoP), the coefficient of variation (CV) and the phase coordination index (PCI) [16,17,18,19,20,21,22,23]. Among these indices, plantar pressure and PCI have shown to be relatively more sensitive measures in analyzing the bilateral coordination or asymmetry of locomotion and balance, which are meaningful variables [16,17,24,25]. According to previous studies, the wearable gait analysis system [26], such as with insole pressure sensors, can analyze gait parameters including gait velocity, cadence, stride length, step length, stride time, single limb support and stance (coordination function) by analyzing pressure between the foot plantar surface and the shoe insole. Therefore, this study developed an insole-type wearable pressure sensor.

In several other previous studies, the insole-type pressure sensor and smart shoes were developed for gait analysis and smart phone applications enabled real-time monitoring of the activities being carried out [27,28]. However, these devices are expensive, and the bio-signals collected and analyzed were reported to be not as accurate for use in clinical research [29]. To solve the cost-related problem of the sensors, researchers tried to apply pressure sensors for a gait analysis system, but they were found to be short-lived, making them still expensive for both users and researchers. Based on these advantages, researchers are conducting studies on an insole type textile pressure sensor considering the properties of conductive textile that is user friendly and inexpensive [30,31,32,33,34]. However, studies on the clinical application of the wearable textile insole sensor are scarce. 

Therefore, this study developed a wearable textile capacitive pressure-sensing insole to test its feasibility in analyzing hemiparetic gait patterns and distinguishing its characteristics from normal gait. 

## 2. Materials and Methods

### 2.1. Textile Capacitive Pressure Sensing Insole (TCPSI) 

**Figure 1:**
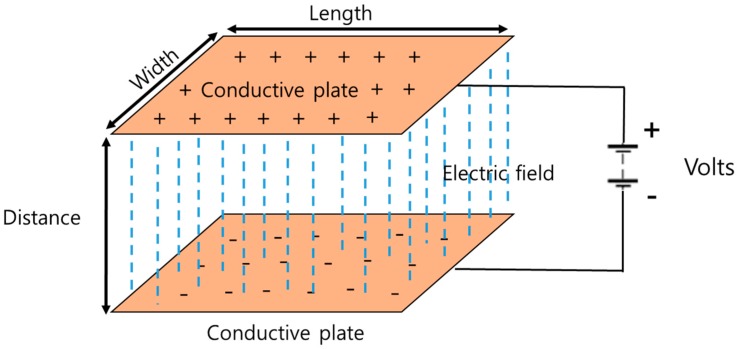
The structure of parallel capacitor used for the textile capacitive pressure sensing insole (TCPSI).

The textile capacitive pressure sensing insole (TCPSI) was designed based on Coulomb’s law as shown in Figure 1. The capacitance is determined by how much electricity is collected between the two conductive plates. The wider the gap between the plates, the higher the capacitance. 1 Farad (F) is equal to the capacitor charged at 1 C when a voltage of 1 V is applied. Equation (1) is a formula for calculating the parallel capacitance value, and the C value is derived using the above formula.

C = *Q*/*V* = *ε*(*A*(width × length))/*d* (1)

where, *d* means the distance between two plates, *A* means the area of plates (width × length), *ε* is the permittivity material between the plates.

**Figure 2:**
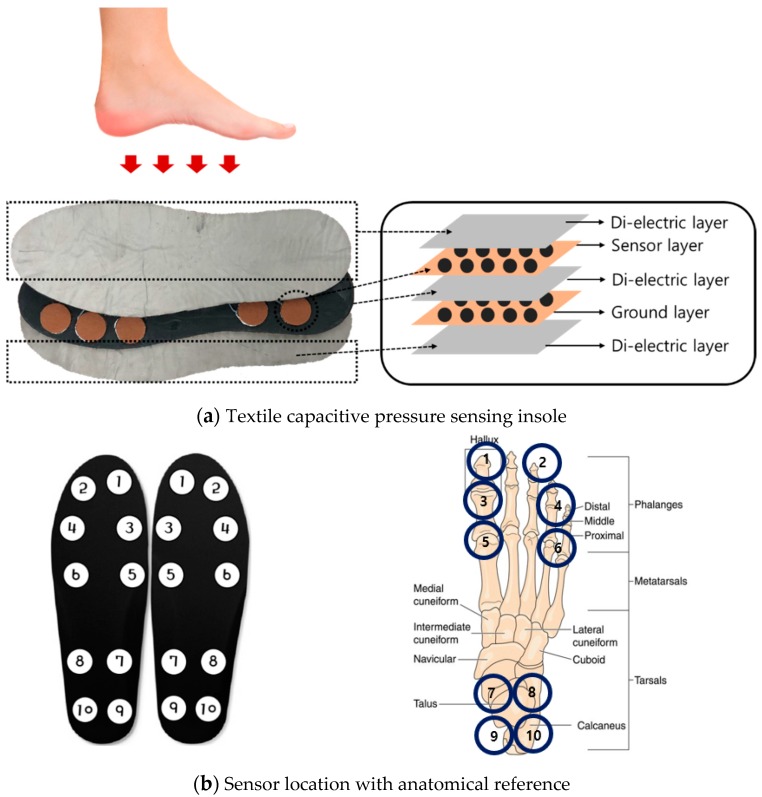
The structure of the textile capacitive pressure sensing insole (TCPSI) and sensor location, (**a**) textile capacitive pressure sensing insole, (**b**) sensor location.

For TCPSI, a parallel capacitance measurement method was applied as illustrated in Figure 2a. The W-290-PCN model (Ajin Electronics, Gangseo, Busan, ROK) conductive textile was used and its characteristics have been described in Table 1. The textile consists of polyester, sequentially plated with nickel, copper, and nickel. Non-conductive rubber with a thickness of 3 mm was placed between a sensor layer and a ground layer, and another two layers of non-conductive woven textiles were placed on the top and bottom of all other layers as a cover and shock absorber. Figure 2b shows the size (2 × 2 cm^2^) and locations of the 10 channels in each insole. Sensor channel 1 was located in the medial upper corner of the insole to detect the pressure mainly under the big toe area (1st, 2nd distal phalanges) and channel 2 was located in the lateral upper corner of the insole to detect the pressure under the 3rd 4th, and 5th distal phalanges. These channels were to detect the plantar pressure in the forefoot during the pre-swing and toe-off phases. Channels 3 and 4 were located below the channels 1 and 2 to detect the pressure under the proximal interphalangeal joint area. Channels 5 and 6 were located in the metatarsal-phalangeal joint area. Channels 7, 8, 9 and 10 were located in the heel area.

### 2.2. Gait Monitoring System

**Figure 3:**
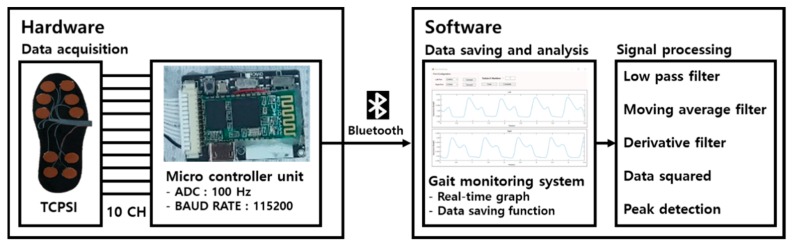
The schematic diagram of the gait monitoring system.

Figure 3 shows the system architecture of our proposed gait monitoring system. The hardware collects data from TCPSI and transfers them to the software via Bluetooth communication. The software receives, saves, and analyzes the data using the C#-based gait monitoring system and matlab2018a.

#### 2.2.1. Hardware

For the micro controller unit (MCU), the STM32F103 model developed by STMicroelectronics, USA was used. The response time of MCU was set up at 100 ms and the operation voltage was 3.7 V. The MCU pre-scaler was set up at 3 and the period was 400 based on 72 MHz main clock frequency. Based on this, the sensing period was calculated and it was set at 16 us by using I^2^C communication. 

For the capacitance measurement function, the MPR121QR2 sensor (Freescale Inc., Austin, TX, USA) was used; it converts capacitance to digital values. This sensor was used in our previous study [35] testing the feasibility of our capacitive insole sensor and showed a high correlation (R^2^ > 0.90, *p* < 0.05) with F-scan (Tekscan Inc., Boston, MA, USA). The MPR121QR2 sensor has a resolution of 0.01 pF and the measurement range covers 10 pF to 2000 pF. The data were sampled at 100 Hz [36]. Normalization was performed for all 10 channels of the insole sensor before signal processing. The standard deviation values between the data collected from the 10 channels were lower than 0.03 pF. 

The HM-13 chip was used for Bluetooth communication and the baud rate was set to 115,200, the non-parity bit was 0, stop bit was 1.

**Figure 4:**
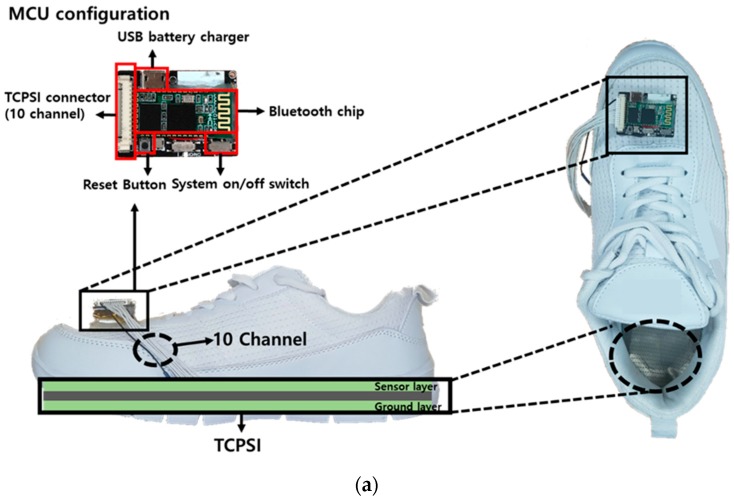

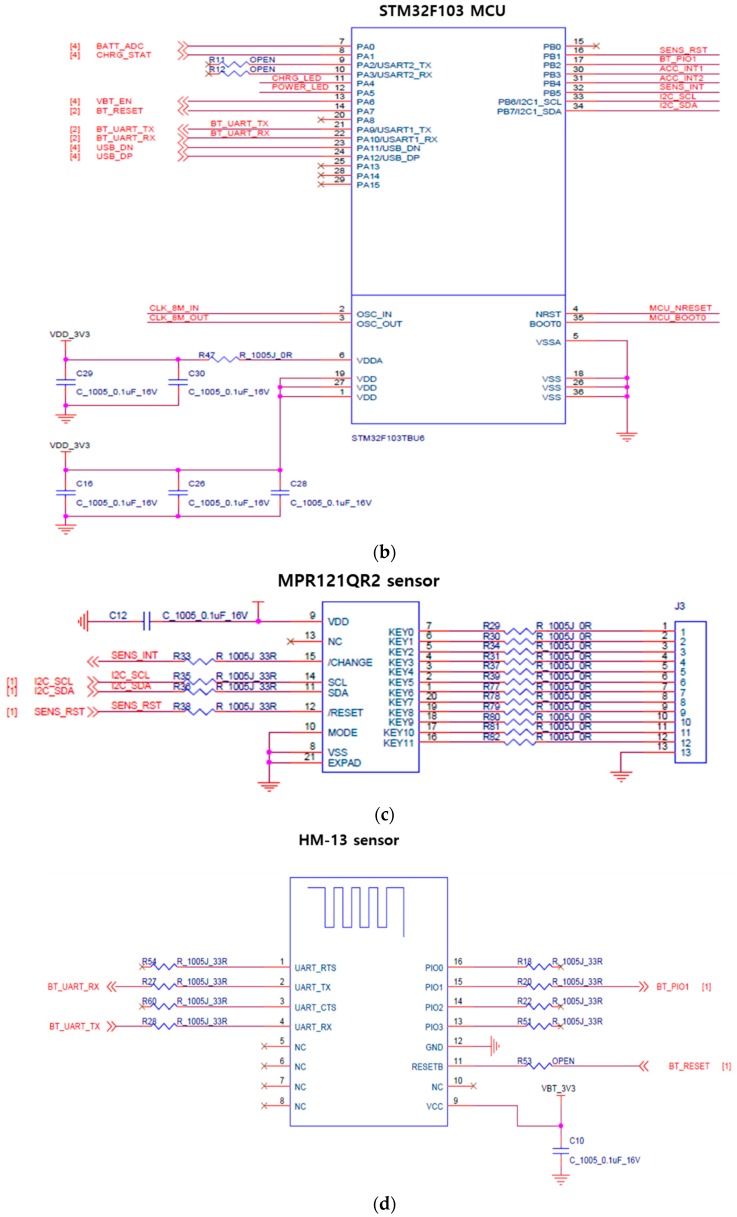
The structure of the printed circuit board (PCB) board, (**a**) PCB configuration, (**b**) STM32F103 MCU, (**c**) MPR121QR2 capacitance to digital converter, (**d**) HM-13 Bluetooth module schematic diagram.

The printed circuit board (PCB) was developed containing all sensors as presented in Figure 4a,b shows the MCU, MPR121QR2, and Bluetooth communication part of the developed PCB schematics. 

#### 2.2.2. Software

A gait monitoring application was designed based on C# language. It visualizes the plantar pressure changes in real time by using capacitance values from the insole sensor. The unit of pressure in our sensing system is capacitance itself, that is, the difference value between pre-pressure (baseline; no pressure applied) and post-pressure at the time of a heel strike (HS) and toe-off (TO). The visualization was to provide feedback on plantar pressure distribution during walking. The software saves the data and exports them in a text file or an excel file. All data are stored as an integer and the software checks in real time whether the data are being received from all 20 channels, 10 channels from each foot. 

**Figure 5:**
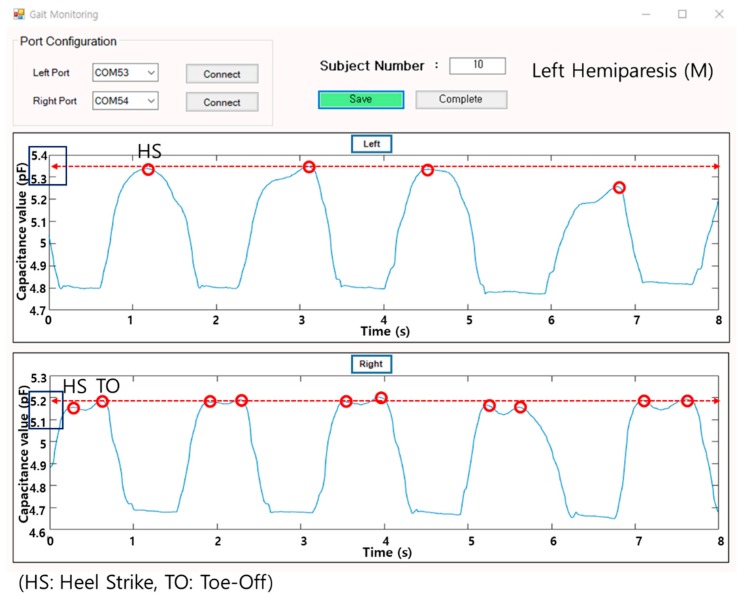
Gait monitoring application (left hemiparetic male patient data sample).

Figure 5 below is showing the gait data of a left hemiparetic male patient. The graph on top presents the real time gait signals, which are based on the capacitance values received from the left foot (insole), and the graph on the bottom visualizes the data from the right foot. Each of the high peaks at the time of the heel strike and toe-off are marked and the average values of the peaks are shown in a dotted line.

### 2.3. Data Analysis

#### 2.3.1. Percentage of Plantar Pressure Difference (PPD)

**Figure 6:**
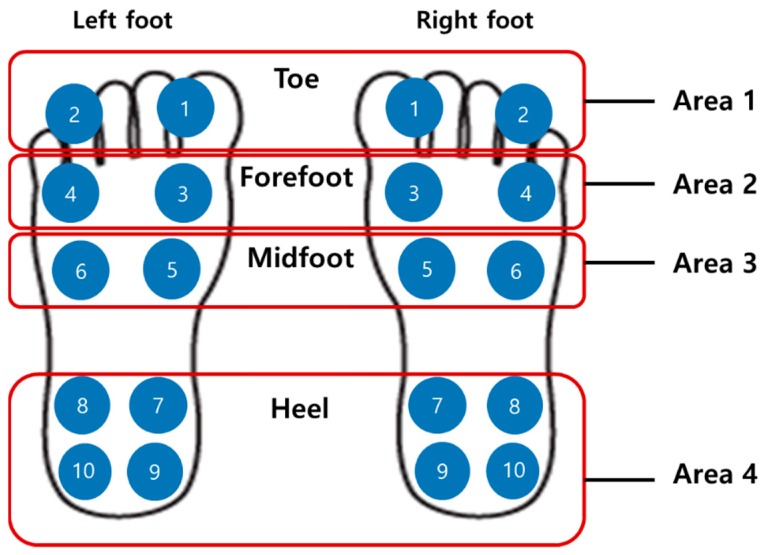
The percentage of plantar pressure difference calculation method diagram.

The PPD was calculated by using Equation (2) as expressed below [17].

(2) $$\mathrm{Percentage}\;\mathrm{of}\;\mathrm{plantar}\;\mathrm{pressure}\;\mathrm{difference}\;\left(\%\right)=\;\frac{2\left|Pressure_{Left}-Pressure_{Right}\right|}{Pressure_{Left}+Pressure_{Right}}\times 100$$

where, $Pressure_{Left}$ and $Pressure_{Right}$ refers to the summed pressure data on each foot as shown in Figure 6. 

#### 2.3.2. Plantar Pressure Distribution Comparison Analysis

**Figure 7:**
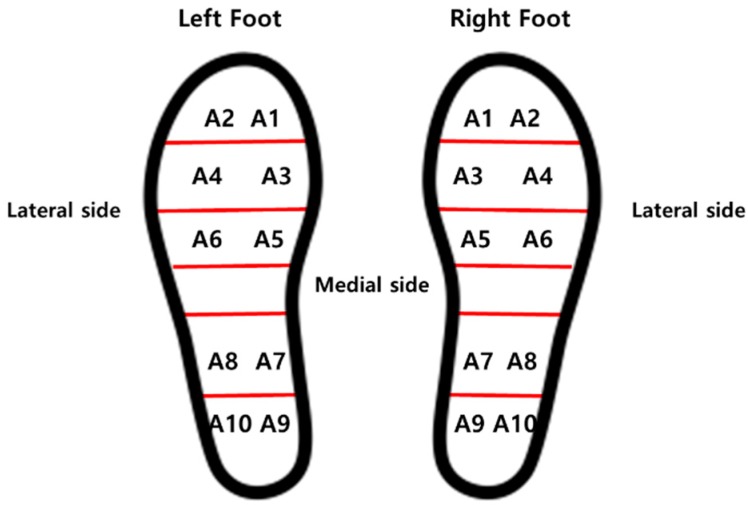
Sensor locations for plantar pressure detection.

For analysis of the weight-shifting patterns during gait, the plantar pressure data received from all 10 sensors on each foot were summed and averaged for medial-lateral and right-left weight shifting characteristics comparison between hemiparetic patients and healthy adults as illustrated in Figure 7.

#### 2.3.3. Signal Processing

**Figure 8:**
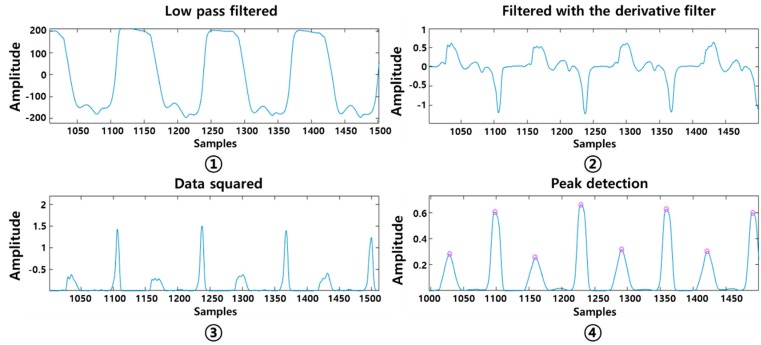
Signal processing order.

Four stages of signal pre-processing were performed consecutively as shown in Figure 8. The low pass filter with a cut-off frequency of 3 Hz was first applied followed by a moving average filter with 5 point. The gait signal was smoothed and noise was removed. The first-order differential filter was then applied to sharpen the gradients on the y-axis, making the high peaks higher and the low peaks lower. This process made the slope of the original signal steeper and more prominent as the change value on the y-axis increased. This study developed an algorithm to detect the highest peaks at the time of the heel strike and toe-off, at an interval of 300 ms. Considering person-to-person gait pattern difference, the interval range was selected based on our previous gait analysis study results using the prototype insole sensor in healthy young adults [35]. The heel strike and toe off were detected within 300 ms intervals.

This was designed based on a local maxima algorithm.

Each initial contact (heel strike) of one side of the leg was marked and calculated for the step count and a stride time was defined by the time between two consecutive heel strikes of the same foot [37].

The coefficient of variation (CV) of the stride time was calculated [38]. 

PCI was calculated by first defining the stride time of one foot as a gait cycle (360°; from one leg’s heel strike to the next heel strike) and the phase (φ ideally 180°; time point of contralateral heel strike). The phase of the ith stride ($\varphi_{i}$, in degrees) is defined by normalizing the step time with respect to the stride time Equation (3). The sum of the CV of phase (*φ*_CV) and the mean absolute difference between phase and 180° $\left(\varphi_{\mathrm{ABS}}\right)$ was defined as PCI [21,22]. $\varphi_{i}$ is an index that evaluates the symmetry of bilateral stepping phases and is the distance between one heel strike and the next of the opposite leg calculated in degrees, ideally 180°  for successful walking. The step time of each leg should normally be equal to the half the gait cycle, but for the asymmetric cases $t_{S_{i}}$ refers to the shorter swing phase and $t_{L_{i}}$ for the longer swing phase; the time of heel strike (of step *i*) during short and long swing phases, respectively. As shown below in the equation, *φ*_ABS indicates the balance between the two feet Equation (4). *φ*_CV refers to the coefficient of variation of $\varphi_{i}$ which represents a consistency of both feet during walking Equation (5).

(3) $$\varphi_{i}=360{}^{\circ}\;\times \;\frac{t_{S_{i}}\;-\;t_{L_{i}}}{t_{L\left(i+1\right)}-t_{L_{i}}}$$

(4) $$\varphi \_ABS=\frac{\varphi \_ABS}{180}\;\times 100$$

(5) $$\varphi^{\prime }=\;\frac{1}{N}\;\sum_{i=1}^{n}\varphi_{i}\;\delta =\sqrt{\frac{1}{N}}\overset{n}{\underset{i=1}{\sum }}{\left(\varphi^{\prime }-\;\varphi_{i}\right)}^{2},\;\varphi \_CV=\;\frac{\delta }{\varphi^{\prime }}$$

### 2.4. Experimental Protocol

For this study, a total of 35 subjects were recruited and approved by the institutional review board (IRB No. SCH2016-130). Eighteen hemiparetic patients (12 males, 6 females) with an average age of 63.94 ± 8.75 years and 17 healthy adults (7 males, 10 females) with an average age of 56.47 ± 17.19 years participated. Three of the hemiparetic patients were diagnosed with bilateral hemiparesis, 6 with left hemiparesis, and 9 with right hemiparesis (Table 2 and Table 3). The patients who participated in this study were those diagnosed with a stroke between July 2016 and February 2017. 

The experiment took place at a local hospital under close supervision by a physical therapist. Each participant was asked to walk down a 20-m long corridor back and forth (1 round trip) at a comfortable speed while wearing the given sneakers with TCPSI inside. The TCPSI sensor was prepared according to the subject’s shoe size (all sizes between 235 to 285 mm). Furthermore, TCPSI was developed to fit the size of the shoe and was configured to collect and save the data in real time while the subjects walked at a comfortable speed. For the patient subjects who complained of pain or discomfort during the experiment, a resting time of more than 5 min was given.

**Figure 9:**
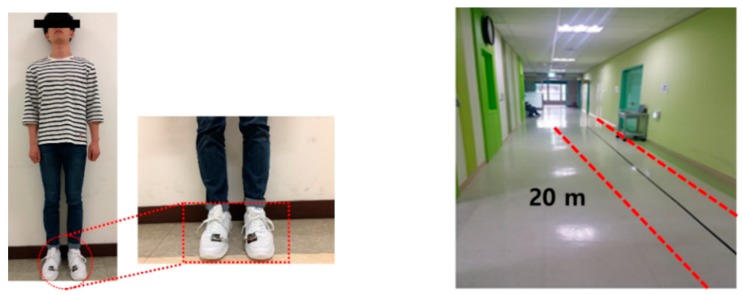
Experimental environment.

The experiment environment is illustrated in Figure 9. All subjects were adequately informed about the experiment procedure and the experiment was conducted after obtaining written consent from all participants.

The collected gait data were analyzed by using the PPD, step count, stride time, CV and PCI of hemiparetic patients and compared their gait coordination functions with those of healthy adults.

## 3. Results

### 3.1. The Results of Plantar Pressure Analysis

#### 3.1.1. Percentage of Plantar Pressure Difference (PPD)

**Figure 10:**
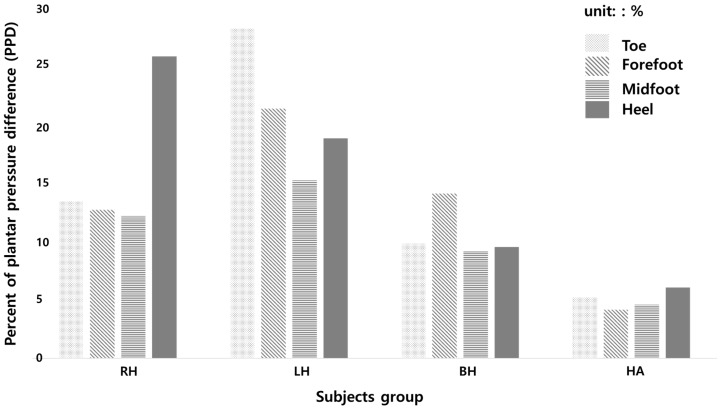
The results of percent of the plantar pressure difference. (RH: right hemiparesis, LH: left hemiparesis, BH: bilateral hemiparesis, HA: healthy adults).

Table 4 shows the percentage of plantar pressure difference in each subject group. The right hemiparesis had PPD of 13.43% in the toe area, 12.74% in the forefoot area, 12.19% in the midfoot area, and 26.73% in the heel area. The left hemiparesis had 28.27%, 21.38%, 15.25% and 18.86%, respectively. The bilateral hemiparesis had 9.91%, 14.10%, 9.17%, 9.52% and healthy adults had 5.26%, 4.15%, 4.59% and 6.06%, respectively. Figure 10 shows the percentage of plantar pressure difference results of each subject group.

#### 3.1.2. Plantar Pressure Comparison Analysis

The average plantar pressure on the left foot (unaffected) of the right hemiparetic patients was 6.11 ± 0.08 pF (capacitance difference value) in the lateral side of the foot and 6.50 ± 0.27 pF in the medial side. The right foot (affected) showed 5.17 ± 0.46 pF and 5.45 ± 0.29 pF, respectively (Table 5). 

Table 6 shows the average plantar pressure of left hemiparetic patients. The lateral side of the left foot (affected) showed 5.11 ± 0.18 pF of pressure and the medial side had 5.01 ± 0.16 pF. The unaffected right foot had 6.14 ± 0.18 pF on the lateral side and 6.14 ± 0.11 pF on the medial side.

In Table 7, the average plantar pressure on the lateral side of the left foot was 5.62 ± 0.33 pF and of the medial side was 5.36 ± 0.38 pF in bilateral hemiparetic patients. The medial side of the right foot showed 5.42 ± 0.57 pF and the lateral side 5.66 ± 0.33 pF. 

Table 8 shows the average plantar pressure of healthy adults. The lateral side of the left foot had 6.39 ± 0.43 pF, the medial side had 6.24 ± 0.38 pF, the lateral side of the right foot had 6.41 ± 0.42 pF, and the medial side had 6.39 ± 0.29 pF.

### 3.2. The Results of Gait Parameter Analysis

#### 3.2.1. Step Count

The average step count during 40 m walk in right hemiparetic patients was 46.83 ± 13.89 steps and for the left hemiparetic patients was 38.44 ± 4.53 as shown in Table 9. The bilateral hemiparetic patients was 40.66 ± 16.77 and healthy adults was 31.29 ± 8.97, respectively.

#### 3.2.2. Stride Time

The average stride time of the left foot in the right hemiparetic patients was 1.66 ± 0.20 s and that of the right foot was slowed to 1.77 ± 0.19 s as shown in Table 10. The average time difference between the two feet was 0.11 ± 0.04 s. 

The average stride time of left hemiparetic patients showed to be slowed to 1.75 ± 0.42 s on the left (affected) side and 1.64 ± 0.40 s on the right foot as shown in Table 11. The time difference between the two feet was 0.14 ± 0.04 s in average. 

The average stride time of the left foot in bilateral hemiparetic patients was 1.48 ± 0.06 s and that of the right foot was 1.52 ± 0.03, and the time difference between the two feet was 0.04 ± 0.04 as illustrated in Table 12.

Table 13 shows the average stride time of healthy adults and the time difference between the two feet. The stride time of the left foot was 1.34 ± 0.12 s and of the right was 1.35 ± 0.12 s. The average stride time difference between the two feet was 0.02 ± 0.03 s.

The stride time CV of each group is presented in Table 14. The right hemiparetic patients had a CV of 13.33 ± 4.52% (left foot) and 23.88 ± 3.29% (right foot). The left hemiparetic patients had 20.83 ± 6.70%, 12.00 ± 3.34%, the bilateral hemiparetic patients had 19.33 ± 2.51% and 18.00 ± 1.73%, respectively. The healthy adults had 2.82 ± 1.91% and 2.47 ± 1.62%, respectively.

**Figure 11:**
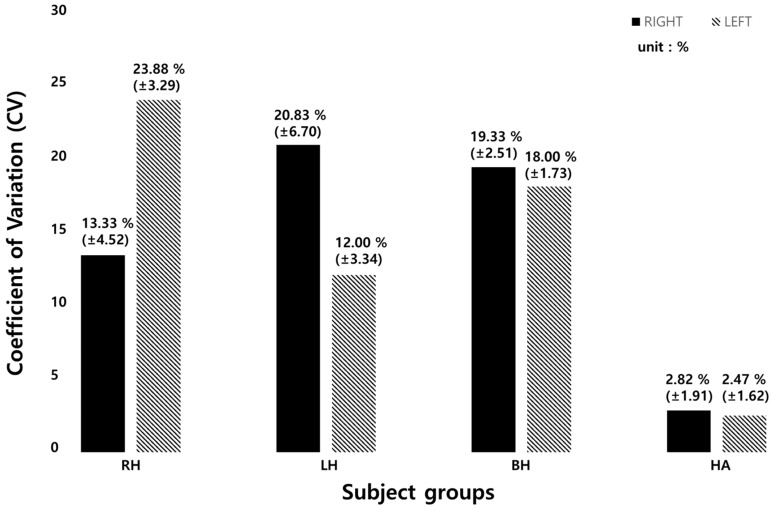
Average stride time CV of each subject group. (RH: right hemiparesis, LH: left hemiparesis, BH: bilateral hemiparesis, HA: healthy adults).

Figure 11 below shows the results of average stride time CV of each subject group. The CV of the left foot in left hemiparetic patient group was 22% and that of the right foot was 12%. The CV of the left foot in right hemiparetic patient group was 13% and that of the right foot was 24%. For bilateral hemiparetic patient group, the CV of the left foot was 19% and that of the right was 18%. The healthy group showed to have a CV of 2% on both sides of the foot.

#### 3.2.3. Phase Coordination Index (PCI)

**Figure 12:**
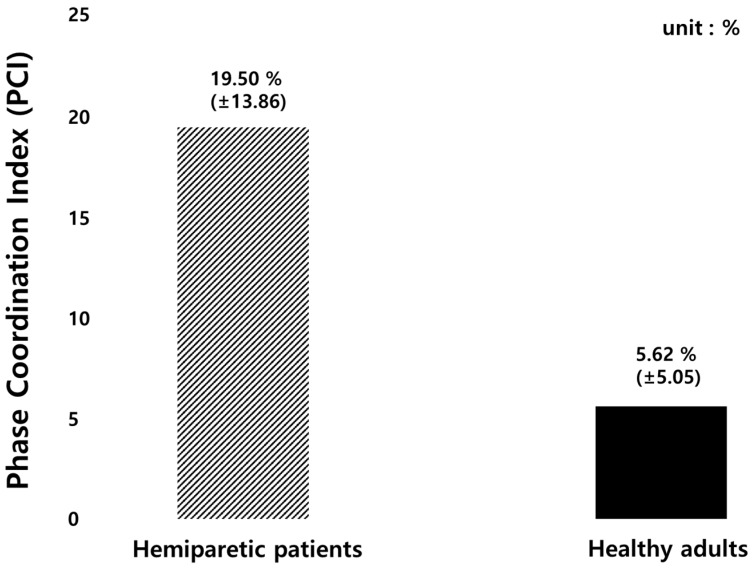
The results of PCI value comparison between hemiparetic patients and healthy adults.

**Figure 13:**
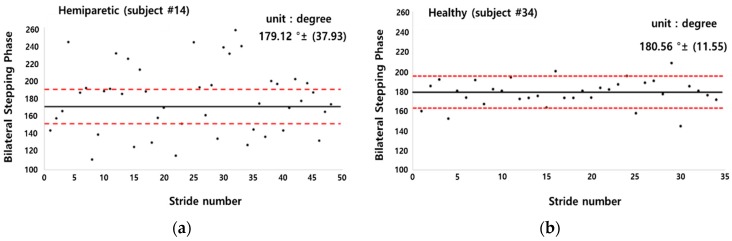
Sample bilateral stepping phase symmetry values plotted based on the data of, (**a**) left hemiparetic subject number 14; (**b**) healthy subject number 34.

The PCI and bilateral stepping phase symmetry were calculated to evaluate gait coordination of hemiparetic patients and healthy adults as illustrated in Figure 12 and Figure 13, respectively. The average value of PCI in hemiparetic patients was 19.50 ± 13.86% and the φ_CV value was 0.20 ± 0.17%, the φ_ABS was 41.09 ± 37.83° and the φ average was 180.88 ± 56.78°. The PCI value of healthy subjects was 5.62 ± 5.06%, the φ_CV was 0.12 ± 0.11%, the φ_CV was 9.89 ± 8.99°, and the φ average was 176.64 ± 13.14° as summarized in Table 15.

Figure 13 shows a sample comparison result in a scatter plot of bilateral stepping phase symmetry between a hemiparetic (subject #14; left hemi) patient and a healthy adult (subject #34). The hemiparetic patient’s bilateral stepping phase was observed to be widely scattered from the standard normal value of 180° compared to that of the healthy subject. 

## 4. Discussion

With the aim of producing a practical and reliable device to quantitatively analyze the human gait, a 10-channeled TCPSI and a real-time monitoring system were developed and applied in this study for hemiparetic gait pattern analysis in comparison with healthy adults. The results of this study showed that our sensor is capable of detecting and distinguishing the differences in plantar pressure (PPD), the step count, the stride time, the coefficient of variation, and the phase coordination index (PCI) between paretic and healthy limbs. These parameters were selected for analysis, because a stroke survivors’ gait abnormality is characterized by a pronounced gait asymmetry [25], decreased gait speed and stance phase, shorter stride length, and prolonged swing phase of the paretic limb [39]. These gait abnormalities along with muscle imbalance and weakness leads to a high risk of falls [40,41]. 

In this study, the subjects with hemiparesis showed the highest PPD in the toe area where the sensor numbers 1 and 2 were located (right hemi: 13.43%, left hemi: 28.27%). The closer to 0% the PPD is, the less the differential pressure of the feet, which means that the feet are well balanced [17]. Sanghan et al. (2015) reported that the PPD of hemiparetic patients was 3 times higher than that of the healthy group [17]. Normally in healthy subjects, the heel contacts first, followed by the midfoot, the forefoot, and then toe pressure. However, since hemiplegic patients do not pressurize the paralyzed feet properly when walking, it may have showed higher pressure in the toe area and in general compared to the healthy adult group. This result was congruent with the results in Perry’s research (1992) [39]. Based on this, our proposed sensor was confirmed that it can detect the dynamic pressure difference of the two feet and be analyzed. In gait analysis, plantar pressure distribution is an important parameter for evaluating the balancing capability in terms of weight-shifting, balance strategies, and a risk of falls.

The plantar pressure distribution comparison analysis showed that the plantar pressure moves towards the lateral side of the affected foot in hemiparetic patients. This phenomenon occurs as a form of compensation to avoid falling. The patients shift their bodyweight towards the unaffected side for more secured stability [42]. The plantar dynamics of the involved leg exhibited a transfer of initial contact from the hind to the forefoot, increased lateral plantar support, limited roll-over, and reduced or absent push-off at a terminal stance. There was a tendency for heel support to disappear on the paretic side with reduced functional abilities.

Field et al. (2013) reported that the patients with a stroke reduce daily stepping activities by 27% compared to a healthy adult [43]. This was also found in our study results. The step count of hemiparetic patients was higher than that of healthy adults.

The average stride time difference between the right and left foot of left hemiparesis was 0.14 ± 0.04 s (Table 4), those of the right hemiparesis was 0.11 ± 0.04 s, and of the bilateral hemiparesis was 0.04 ± 0.04 s, respectively (Table 5 and Table 6). The temporal aspects of hemiplegic gait are characterized by increased stride times [44]. Hemiplegic patients usually have reduced joint excursion and insufficient forward propulsion, which may lead to an asymmetrical and unstable walking pattern [5]. Since the single support time of the affected limb is significantly shorter than that of the unaffected limb, the unaffected limb’s step length is shorter than the affected limb’s step length [4,6]. Likely, the stride time of the affected foot was longer than the unaffected side in our study. 

As presented in Table 6, the time difference in the average stride time in healthy adults was 0.02 ± 0.01 s and the average stride time difference was three times lower than the hemiparetic patients. Mackenzie et al. (2006) and Sartini et al. (2010) reported that the stride time was related to falling and an abnormal gait pattern causes a risk of falls [45,46]. The elderly who have experienced falls are associated with a decrease in walking speed, stepping and an increase in step symmetry [47]. Our proposed sensor system can also detect and decrease the risk of falls by real-time gait monitoring.

The CV is used to compare the magnitude of the change regardless of the data dimension. In this study, because the step length and the stride time may be different depending on the subject and the degree of the disease, the CV was calculated and the change amount was examined. The CV of stride time with heathy adults was 5 times higher than hemiparetic patients as shown in Table 8 and Figure 9. This means that hemiparetic patients cannot walk with a constant gait speed and thus walk irregularly. It was confirmed that our sensor can analyze various gait parameters.

This study performed the PCI comparison between hemiparetic patients and healthy adults because hemiparetic gait is characterized by mild to severe asymmetric patterns. Hemiparetic patients have increased stance phase and double support duration. In addition, the stride length and step length decrease, and the paralyzed side has longer step [48]. The PCI is an indicator for evaluating the balance of a pair of feet. A value closer to 0% refers to a higher balance between the two feet [23,24,25,48]. The PCI was originally developed to evaluate the asymmetry during walking and many studies evaluated the gait asymmetry of patients with Parkinson’s disease and a stroke [23,24,25,48]. In the previous studies evaluating gait asymmetry in patients with stroke patients [48], the PCI value of stroke patients was 19.5% ± 2.3% and that of healthy subjects was 6.2% ± 1.0%. The PCI value was approximately 3 times higher in the patient group than in the healthy group. 

In this study, the PCI value of stroke patients was 19.5% ± 13.9% and that of healthy subjects was 5.6% ± 5.1%, showing similar results to the previous studies as summarized in Table 7. Our results showed a higher standard deviation of the PCI value than that of previous studies. This may be because the age range of the subjects participating in this study was larger (63.3 ± 8.6 yrs.) The ages of the subjects (number 2, 6, 8, 10, and 14) were 65, 63, 63, 60, and 68, respectively, and were diagnosed with hemiparesis. The PCI values of the five subjects were 4.07%, 4.00%, 4.64%, 0.98%, 4.03%, respectively, which seemed to be similar to those of healthy adults. According to the previous studies, aging is associated with decreased stride time, velocity, step length during gait. Slowed walking speed leads to larger gait variability [37]. Other previous studies explored age-related changes by analyzing gait speed and PCI values, and found out that the average gait speed decreases and the PCI value increases every decade from the age of 70 years [49,50,51]. 

Study limitations: Generalizing the results of this study may be difficult because the number of subjects was small. In our previous study [35], a soft-material-based pressure insole and its performance feasibility test for performing a clinical experiment was developed and analyzed. The study results showed that our pressure insole sensor was confirmed to have a high correlation with F-scan (R^2^ ≥ 0.90, *p* < 0.05). Therefore, the sensor performance analysis was not repeated in this study.

## 5. Conclusions

The insole-type textile capacitive pressure sensor and a real-time gait monitoring system developed in this study was tested and confirmed for its clinical feasibility in applying the sensor system to hemiparetic patients. Hemiparetic gait patterns were well distinguished from healthy gait. Our proposed sensor may be used as a wearable gait function evaluation tool, external feedback gait training device, and a simple gait pattern analyzer for both hemiparetic patients and healthy individuals.

## Figures and Tables

**Table 1:** Characteristics of the W-290-PCN model.

	Value	Structure
Weight (g/m^2^)	81 ± 5	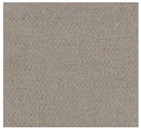 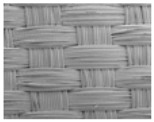
Width (mm)	1100 ± 5	
Density (g/m^3^)	188 ± 5	
Thickness (mm)	0.10 ± 0.01	
Shielding effectiveness (dB)	70	
Surface resistance (Ω)	<0.05	

**Table 2:** General characteristics of hemiparetic subjects.

Subject	Sex	Age	Height	Weight	BMI	Shoe Size	Hemi Side	Onset
1	M	54	174	80	26.42	260	L	12.2016 2016.12
2	M	65	173	74	24.72	270	R	10.2016
3	M	76	170	90	31.14	270	R	11.2016
4	M	59	167	73	26.17	255	L	11.2016
5	M	72	177	75	23.93	275	R	10.2016
6	M	63	172	70	23.66	275	R	12.2016
7	M	75	175	70	22.85	260	L	09.2016
8	M	63	176	64	20.66	275	B	07.2016
9	F	64	150	45	20.00	235	B	12.2016
10	M	60	178	79	24.93	295	R	01.2017
11	M	78	170	67	23.18	265	R	08.2016
12	F	63	163	67	25.21	245	R	01.2017
13	F	60	150	38	16.88	225	L	07.2016
14	M	68	168	58	20.54	260	L	01.2017
15	F	47	175	75	24.48	230	L	01.2017
16	M	49	162	90	34.29	255	B	02.2017
17	F	73	145	50	23.78	250	L	02.2017
18	F	62	150	45	20.00	240	R	12.2016
AVG		63.94	166.39	67.22	24.05	257.78		
SD		8.75	10.69	14.93	4.05	18.08		

RH: right hemiparesis, LH: left hemiparesis, BH: bilateral hemiparesis, HA: healthy adults.

**Table 3:** General characteristics of healthy subjects.

Subject	Sex	Age	Height	Weight	BMI	Shoe Size
1	F	34	168	54	19.13	260
2	F	31	161	48	18.51	235
3	M	27	178	75	23.67	265
4	M	48	168	63	22.32	260
5	M	30	178	70	22.09	265
6	M	45	167	65	23.30	260
7	M	75	175	75	25.95	260
8	F	57	159	54	21.35	235
9	F	68	150	51	22.66	225
10	F	58	163	80	30.11	245
11	M	71	165	68	24.97	250
12	F	71	158	62	24.83	245
13	F	71	157	68	27.58	245
14	F	64	156	54	22.18	230
15	M	77	168	80	28.34	260
16	F	69	155	66	27.47	235
17	F	64	162	72	27.43	240
AVG		56.47	164	65	24.22	247.94
SD		17.19	8.04	10.01	3.26	13.11

**Table 4:** Percentage of the plantar pressure difference (unit: %).

Location	RH	LH	BH	HA
Toe	13.43	28.27	9.91	5.26
Forefoot	12.74	21.38	14.10	4.15
Midfoot	12.19	15.25	9.17	4.59
Heel	25.89	18.86	9.52	6.06
AVG	16.06	20.95	10.68	5.01
SD	6.57	5.49	2.30	0.83

RH: right hemiparesis, LH: left hemiparesis, BH: bilateral hemiparesis, HA: healthy adults.

**Table 5:** Average plantar pressure of right hemiparetic patients (*n* = 9) (unit: pF).

Location	Left Foot				Right Foot			
	Medial Side		Lateral Side		Medial Side		Lateral Side	
	Channel	Pressure	Channel	Pressure	Channel	Pressure	Channel	Pressure
Toe	A2	6.59	A1	6.23	A1	5.63	A2	5.81
Forefoot	A4	6.47	A3	6.10	A3	5.51	A4	5.58
Midfoot	A6	6.14	A5	6.03	A5	5.22	A6	5.56
Heel	A8	6.88	A7	6.04	A7	5.03	A8	5.21
Heel	A10	6.40	A9	6.13	A9	4.47	A10	5.10
AVG		6.50		6.11		5.17		5.45
SD		0.27		0.08		0.46		0.29

**Table 6:** Average plantar pressure of left hemiparetic patients (*n* = 6) (unit: pF).

Location	Left Foot				Right Foot			
	Medial Side		Lateral Side		Medial Side		Lateral Side	
	Channel	Pressure	Channel	Pressure	Channel	Pressure	Channel	Pressure
Toe	A2	5.27	A1	5.41	A1	6.41	A2	6.17
Forefoot	A4	5.01	A3	5.12	A3	6.16	A4	6.21
Midfoot	A6	5.03	A5	4.98	A5	5.95	A6	5.95
Heel	A8	4.87	A7	4.98	A7	6.01	A8	6.19
Heel	A10	4.87	A9	4.94	A9	6.16	A10	6.17
AVG		5.01		5.11		6.14		6.14
SD		0.16		0.18		0.18		0.11

**Table 7:** Average plantar pressure of bilateral hemiparetic patients (*n* = 3) (unit: pF).

Location	Left Foot				Right Foot			
	Medial Side		Lateral Side		Medial Side		Lateral Side	
	Channel	Pressure	Channel	Pressure	Channel	Pressure	Channel	Pressure
Toe	A2	5.51	A1	5.18	A1	4.68	A2	6.07
Forefoot	A4	5.96	A3	5.40	A3	6.20	A4	5.16
Midfoot	A6	5.13	A5	5.64	A5	5.12	A6	5.60
Heel	A8	5.03	A7	5.96	A7	5.56	A8	5.67
Heel	A10	5.17	A9	5.91	A9	5.52	A10	5.82
AVG		5.36		5.62		5.42		5.66
SD		0.38		0.33		0.57		0.33

**Table 8:** Average plantar pressure of healthy adults (*n* = 17) (unit: pF).

Location	Left Foot				Right Foot			
	Medial Side		Lateral Side		Medial Side		Lateral Side	
	Channel	Pressure	Channel	Pressure	Channel	Pressure	Channel	Pressure
Toe	A2	6.61	A1	6.25	A1	6.66	A2	6.34
Forefoot	A4	6.43	A3	6.60	A3	6.30	A4	6.20
Midfoot	A6	5.65	A5	5.83	A5	5.72	A6	6.08
Heel	A8	6.12	A7	6.30	A7	6.67	A8	6.52
Heel	A10	6.41	A9	6.99	A9	6.68	A10	6.83
AVG		6.24		6.39		6.41		6.39
SD		0.38		0.43		0.42		0.29

**Table 9:** The results of step count detection in hemiparetic patients and healthy adults.

Group	Step Count
RH	46.83 ± 13.89
LH	38.44 ± 4.53
BH	40.66 ± 16.77
HA	31.29 ± 8.97

**Table 10:** Average stride time of right hemiparetic patients (*n* = 9) (unit: seconds).

Subject No.	Left Foot	Right Foot	Time Difference (Right–Left)
Subject 2	1.39	1.48	0.09
Subject 3	1.75	1.88	0.13
Subject 5	1.51	1.63	0.12
Subject 6	1.71	1.81	0.10
Subject 10	1.86	1.93	0.07
Subject 11	1.98	2.05	0.07
Subject 12	1.56	1.75	0.19
Subject 16	1.40	1.52	0.12
Subject 18	1.78	1.94	0.16
AVG	1.66	1.77	0.11
SD	0.20	0.19	0.04

**Table 11:** Average stride time of left hemiparetic patients (*n* = 6) (unit: seconds).

Subject No.	Left Foot	Right Foot	Time Difference (Left–Right)
Subject 1	1.58	1.46	0.12
Subject 4	1.39	1.48	0.09
Subject 7	1.54	1.40	0.14
Subject 13	1.65	1.42	0.23
Subject 14	2.58	2.46	0.12
Subject 17	1.79	1.62	0.17
AVG	1.75	1.64	0.14
SD	0.42	0.40	0.04

**Table 12:** Average stride time of bilateral hemiparetic patients (*n* = 3) (unit: seconds).

Subject No.	Left Foot	Right Foot	Time Difference
Subject 8	1.44	1.54	0.10
Subject 9	1.46	1.48	0.02
Subject 15	1.56	1.54	0.02
AVG	1.48	1.52	0.04
SD	0.06	0.03	0.04

**Table 13:** Average stride time of healthy adults (*n* = 17) (unit: seconds).

Subject No.	Left Foot	Right Foot	Time Difference (Right–Left)
Subject 19	1.21	1.21	0.09
Subject 20	1.60	1.60	0.13
Subject 21	1.32	1.32	0.00
Subject 22	1.25	1.25	0.00
Subject 23	1.27	1.27	0.00
Subject 24	1.18	1.18	0.00
Subject 25	1.29	1.32	0.03
Subject 26	1.27	1.27	0.00
Subject 27	1.41	1.41	0.00
Subject 28	1.34	1.33	0.01
Subject 29	1.39	1.38	0.01
Subject 30	1.49	1.49	0.00
Subject 31	1.43	1.44	0.01
Subject 32	1.17	1.18	0.01
Subject 33	1.34	1.42	0.08
Subject 34	1.60	1.60	0.00
Subject 35	1.36	1.35	0.01
AVG	1.34	1.35	0.02
SD	0.12	0.12	0.03

**Table 14:** Coefficient of Variation (CV) of stride time in each group (unit: %).

Variables	Left Foot	Right Foot
RH	13.33 ± 4.52	23.88 ± 3.29
LH	20.83 ± 6.70	12.00 ± 3.34
BH	19.33 ± 2.51	18.00 ± 1.73
HA	2.82 ± 1.91	2.47 ± 1.62

RH: right hemiparesis, LH: left hemiparesis, BH: bilateral hemiparesis, HA: healthy adults.

**Table 15:** The results of the phase coordination index (PCI) detection.

Variables	Hemiparetic (*n* = 18)	Healthy (*n* = 17)
Phase Coordination Index (%)	19.50 ± 13.86%	5.62 ± 5.05%
φ_CV (%)	0.20 ± 0.17%	0.12 ± 0.11%
φ_ABS (deg°)	41.09 ± 37.83°	9.89 ± 8.99°
φ (deg°)	180.88 ± 56.78°	176.64 ± 13.14°
